# Effects of mobile health technology on physical activity in pregnant women: a systematic review and meta-analysis

**DOI:** 10.1186/s12884-025-08455-6

**Published:** 2025-11-21

**Authors:** Jiayi Yao, Haozhe Wang, Shiguan Jia, Wenjia Chen, Enliang Hu

**Affiliations:** 1 School of Physical Education, China University of Mining and Technology, Xuzhou, 221116 China; 2 Civil Aviation Flight University of China, Deyang, 618307 China

**Keywords:** Mobile health, Pregnancy, Physical activity, Moderate-to-vigorous physical activity, Meta-analysis, Randomized controlled trial

## Abstract

**Objective:**

To systematically evaluate the effectiveness of mobile health (mHealth) interventions on physical activity in pregnant women and to explore key factors influencing the intervention outcomes.

**Methods:**

A systematic search was conducted across databases including PubMed, Web of Science, MEDLINE, Embase, Cochrane Library, CINAHL, Scopus, CNKI, WanFang, and VIP to identify randomized controlled trials (RCTs) assessing the impact of mHealth interventions on physical activity during pregnancy. Study quality was assessed using the Cochrane Risk of Bias tool 2.0 (RoB 2.0). Meta-analysis, including the pooling of effect sizes (using Standardized Mean Difference, SMD) and prespecified subgroup analyses, was performed using RevMan 5.4 software. Due to the limited number of included studies, exploratory meta-regression was conducted as a supplementary analysis and its results were interpreted with caution. No formal statistical tests for publication bias were performed.

**Results:**

Sixteen studies were included in the qualitative synthesis, of which ten studies (1,615 pregnant women) were included in the meta-analysis. mHealth interventions significantly improved total physical activity (TPA) (SMD = 0.44, 95% CI: 0.31 to 0.57, *P* < 0.00001, I² = 0%) and also demonstrated a small but significant improvement in moderate-to-vigorous physical activity (MVPA) (SMD = 0.21, 95% CI: 0.03 to 0.40, *P* = 0.03, I² = 36%). Prespecified subgroup analysis revealed that the gestational week at the start of the intervention was a key factor influencing the effect on MVPA, with interventions initiated in early pregnancy (≤ 14 weeks) showing significantly better outcomes than those started later. In contrast, the effect on TPA was robust across all subgroups.

**Conclusion:**

mHealth interventions effectively promote physical activity in pregnant women, yielding a moderate and robust improvement in total physical activity and a small but significant increase in MVPA. The findings suggest that initiating interventions in early pregnancy (≤ 14 weeks) may be more effective for promoting MVPA. These findings provide evidence-based support for integrating mHealth strategies into routine antenatal care to promote physical activity.

**Systematic review registration:**

This systematic review and meta-analysis has been registered with PROSPERO (www.crd.york.ac.uk/prospero), identifier CRD420251107861.

**Supplementary Information:**

The online version contains supplementary material available at 10.1186/s12884-025-08455-6.

## Introduction

Adequate physical activity during pregnancy is crucial for maternal and infant health [1], not only reducing the risk of complications such as gestational diabetes and pre-eclampsia [2] but also helping to manage weight gain and improve mental health [3]. The World Health Organization recommends that healthy pregnant women engage in at least 150 min of moderate-intensity aerobic exercise per week [4, 5]. However, physical activity levels among pregnant women are generally low worldwide [6], with a systematic review showing that over half of the studies reported low physical activity levels during pregnancy [7]. In China, only about 20% of pregnant women meet the recommended standard [8].

Traditional health interventions face limitations in improving physical activity among pregnant women, such as poor adherence [9] and a low degree of individualization [10]. With the proliferation of mobile technology, mobile health (mHealth) interventions offer a new avenue to address these issues. Through applications and wearable devices, mHealth enables personalized guidance and real-time monitoring [11, 12] and has shown broad application prospects in chronic disease management [13] and health behavior promotion [14]. Evidence suggests that mHealth interventions have the potential to improve glycemic control [15] and reduce adverse pregnancy outcomes [16]. However, the implementation of mHealth interventions also faces challenges. For instance, adherence may decline as gestation progresses [17], and the long-term effects are difficult to sustain after the intervention ceases [18], highlighting the importance of continuous management.

Although mHealth holds great potential for promoting physical activity [19], a critical evidence gap remains, as there is a lack of systematic evidence-based evaluations specifically assessing the impact of mHealth interventions on physical activity in all healthy pregnant women. Existing reviews are either too broad in scope, covering various eHealth interventions [20], or focus primarily on weight management [21], failing to conduct an in-depth analysis of physical activity as a specific outcome [22]. Notably, a recent meta-analysis targeting overweight and obese pregnant women found no significant effect of mHealth on physical activity [23], but it remains unknown whether this applies to healthy pregnant women across all BMI ranges. Therefore, there is an urgent need for a comprehensive assessment that not only examines the impact of mHealth on overall physical activity in healthy pregnant women but also differentiates between activity intensities and explores key factors influencing intervention effectiveness.

Therefore, this study employs a systematic review and meta-analysis to comprehensively evaluate the impact of mHealth interventions on physical activity in pregnant women, aiming to provide scientific evidence for developing evidence-based strategies to promote physical activity during pregnancy.

## Materials and methods

This study was designed and reported in accordance with the PRISMA [24] and Cochrane Handbook [25] guidelines. The study protocol was pre-registered in the PROSPERO International Prospective Register of Systematic Reviews before its initiation (Registration No: CRD420251107861).

### Literature search and study selection

A systematic literature search was conducted across ten electronic databases (PubMed, Web of Science, Scopus, MEDLINE, Embase, Cochrane Library, CINAHL, CNKI, WanFang, and VIP) from their inception to September 4, 2025. Search terms were developed based on the PICOS framework (Population, Intervention, Comparison, Outcome, Study Design) and categorized into population (“pregnant women,” etc.), intervention (“mHealth,” etc.), comparison (“usual care,” etc.), and outcome (“physical activity,” etc.). An example of the search strategy for the PubMed database is provided in Table 1.

**Table 1:** PubMed database literature search strategy

Search Step	Search Terms	Search Field
1	Pregnan OR gestation OR prenatal OR antenatal OR antepartum OR maternal OR(expectant mother)OR (expectant women) OR gravid OR maternit	Title, Abstract
2	Mhealth OR e-health OR (digital health) OR (electronic health) OR eHealth OR m-health OR (mobile health) OR web OR internet OR online OR DVD-based OR smartphone OR (mobile phone) OR wearable OR (social media) OR computer OR (video gaming) OR app OR email OR telemedicine OR e-learning OR elearning OR texting OR SMS OR (text messaging) OR (digital platform) OR (fitness tracker) OR (activity tracker) OR (actigraphy) OR (acceleromet)	Title, Abstract
3	Sport OR (physical activit) OR exercise OR aerobic OR outdoor OR playground OR active recreation OR walk OR bicycl OR biking OR crawl OR swim OR soccer OR Dancing OR jumping OR jogging OR running OR game	Title, Abstract
4	Randomized controlled trial OR controlled clinical trial OR randomized OR randomly OR trial OR control group OR intervention study OR clinical trial OR RCT	Title, Abstract
5	#1 AND #2 AND #3 AND #4	

### Study selection criteria

Inclusion and exclusion criteria were established based on the PICOS framework. (1) Population: Healthy pregnant women aged 18 years or older with a singleton pregnancy and no major pregnancy complications. (2) Intervention: Interventions primarily utilizing mHealth technologies, including but not limited to mHealth applications, wearable device monitoring, SMS reminder systems, and online health platforms. (3) Comparison: Control groups receiving usual antenatal care, wait-list control, or other standard care measures. (4) Outcomes: Studies reporting physical activity duration-related metrics, including total physical activity (TPA) time and moderate-to-vigorous physical activity (MVPA) time, with units standardized to hours/week. TPA refers to the cumulative weekly duration of all-intensity (light, moderate, vigorous) physical activity, reflecting overall activity levels. MVPA time refers to the cumulative weekly duration of activities of moderate intensity (increased heart rate, slightly faster breathing) and above. (5) Study Design: Randomized controlled trials (RCTs). (6) Language: Literature published in Chinese or English. Exclusion criteria were: (1) studies involving multiple pregnancies; (2) participants with high-risk pregnancy conditions, including but not limited to pre-gestational diabetes, chronic hypertension, pre-eclampsia/eclampsia, fetal distress, or a history of severe obstetric complications; (3) presence of severe pregnancy complications such as severe GDM or pregnancy complicated by heart/chronic kidney disease; (4) studies not reporting physical activity duration outcomes or with incomplete data; (5) non-RCT designs; (6) conference abstracts, case reports, and review articles; (7) secondary analyses or overlapping publications from the same RCT. When multiple publications from the same trial were identified, only the primary publication with the most complete physical activity outcome data was included to avoid double-counting participants.

### Data extraction and Preparation

A customized data extraction tool was designed in Excel (Microsoft Inc., Redmond, WA, USA). To ensure accuracy in deduplication, search results from multiple databases were first merged and deduplicated using EndNote X9 reference management software. This was followed by manual verification to remove all remaining duplicates. Subsequently, titles/abstracts and full texts were screened. During the full-text screening, duplicate publications of the same study data were further excluded based on exclusion criterion (7) to ensure that only one publication with the most complete data was included for each independent study, thus preventing duplicate data from biasing the meta-analysis results. Two researchers independently extracted data and cross-verified them to ensure accuracy and completeness. Discrepancies were resolved by a third researcher through arbitration. Extracted data included basic study characteristics (author, publication year), population characteristics (sample size, age, mean BMI), intervention characteristics (type, content, duration), and outcome characteristics (metrics, measurement methods, tool assessment content, and validation status). For studies with incomplete reporting, we attempted to contact the authors for missing data. All data were double-entered and cross-verified to minimize human error and enhance the reliability of the study.

### Quality assessment

The quality of the included studies was assessed using the Cochrane Risk of Bias tool 2.0 (RoB 2.0), which covers five core domains: bias arising from the randomization process, bias due to deviations from intended interventions, bias due to missing outcome data, bias in measurement of the outcome, and bias in selection of the reported result. Each domain was classified as low risk, some concerns, or high risk according to Cochrane standards. Two independent researchers completed the assessment, with disagreements resolved through discussion or consultation with a third expert.

### Statistical analysis

Meta-analysis was conducted using Review Manager 5.4 software. Due to the use of different physical activity assessment tools across studies, the Standardized Mean Difference (SMD) was chosen as the effect size measure, with 95% confidence intervals (95% CI) calculated. Heterogeneity was assessed using the I² statistic and the Q test, with I² values of 25%, 50%, and 75% representing low, moderate, and high heterogeneity, respectively. A random-effects model was used when I² >25% and *P* < 0.10; otherwise, a fixed-effect model was employed. Based on theoretical frameworks and prior evidence, three prespecified subgroup analyses were conducted to explore potential effect modifiers: (1) Intervention Duration: based on a systematic review by Currie et al. (2013) [3], which showed significant variation in intervention duration and noted higher dropout rates in longer interventions. (2) Gestational Week at Intervention Start: based on ACOG guidelines [26] and a review by Rodrigues (2024) [27], which support the benefits of starting exercise in the first trimester. (3) Outcome Measurement Tool: based on a systematic review by Prince et al. (2008) [28], which found systematic differences between subjective and objective measures.

A leave-one-out analysis was performed to assess the influence of individual studies on the pooled effect size and test the robustness of the results. Given the limited number of included studies (fewer than 10 for each outcome), meta-regression was conducted only as an exploratory supplement, with full acknowledgment of the risks of insufficient power and overfitting. Therefore, these results are for reference only and not considered primary conclusions. The main findings are based on the prespecified subgroup analyses. Due to the limited number of studies, formal statistical tests for publication bias were not performed (as the Cochrane Handbook advises against funnel plots and Egger’s test for < 10 studies). Considering the exploratory nature of the study and sample limitations, no adjustments for multiple comparisons were made, but these limitations were fully considered in the interpretation of the results. All prespecified subgroup analysis results should be interpreted with caution.

## Results

### Literature search

**Fig. 1:**
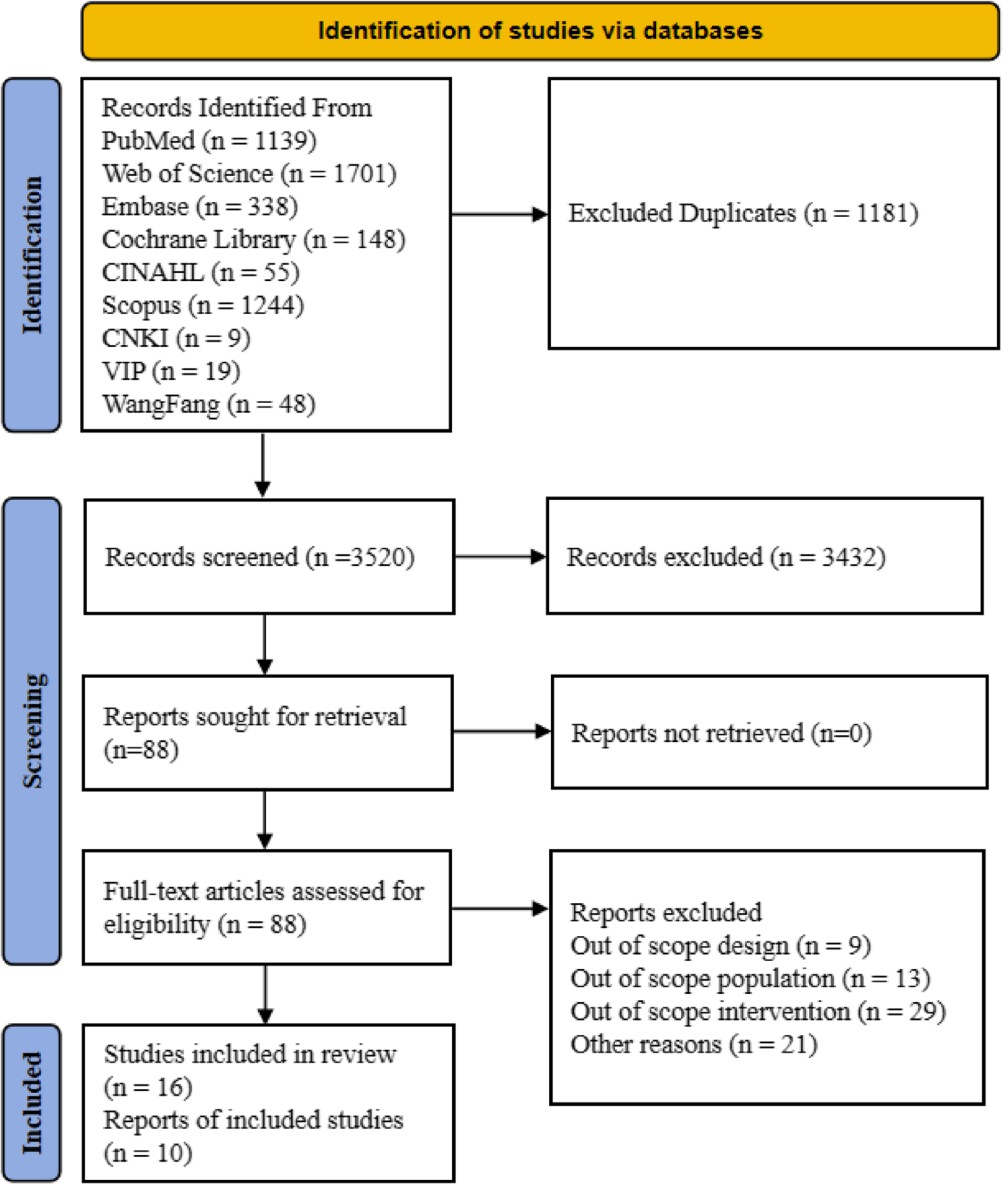
Preferred reporting items for systematic reviews and meta-analysis (PRISMA) study flow diagram [24]

We systematically searched PubMed, Web of Science, Scopus, Embase, Cochrane Library, CINAHL, and three Chinese databases (CNKI, WanFang, VIP), with the search conducted up to September 4, 2025. The initial search yielded 4,701 articles. After removing 1,181 duplicates, we screened the titles and abstracts of the remaining 3,520 articles. Based on the prespecified inclusion and exclusion criteria, 3,432 irrelevant articles were excluded. Subsequently, we assessed the full texts of the remaining 88 reports for eligibility, of which 72 were excluded for not meeting the criteria. Ultimately, 16 studies were included in the systematic review, with 10 of these providing complete quantitative data on physical activity for inclusion in the meta-analysis. The detailed screening process and results are presented in Fig. 1.

### Characteristics of included studies

A total of 16 RCTs were included in the systematic review [19, 21, 29–42], published between 2014 and 2025 [37, 41]. The total sample size was 2,050 pregnant women, with individual study sample sizes ranging from 30 to 565 [29, 40]. Ten of these studies, comprising 1,615 pregnant women, were included in the meta-analysis for reporting complete quantitative data on physical activity duration [21, 30–38] (see Supplementary Table 1). Regarding population characteristics, 12 studies reported BMI data, with mean BMI ranging from 24.0 to 31.0 kg/m² [32, 37]; four studies did not report specific BMI values [19, 21, 31, 39]. There was considerable variation in the timing and duration of interventions. Interventions started between 8 and 20 weeks of gestation [19, 30], with six studies initiating the intervention before 14 weeks [31, 33, 35–37, 42]. Intervention duration ranged from 12 to 26 weeks, with five studies lasting approximately 12 weeks [29, 31, 33, 37, 40] and four lasting about 23 weeks [21, 30, 32, 39]. Ten studies continued until after 32 weeks of gestation or until delivery [19, 21, 30, 32, 34–39]. In terms of intervention content, the interventions in all 16 studies were described and standardized based on the Behavior Change Technique Taxonomy v1 (BCTTv1) [43]. The most widely applied BCTs included: goal setting (BCT 1.1) in 10 studies [21, 30, 32, 33, 36–41], self-monitoring (BCT 2.3) in 9 studies [21, 30, 32, 36–41], personalized feedback (BCT 2.2) in 7 studies [21, 30, 33, 39–41], and problem-solving (BCT 1.2) in 2 studies [36, 40]. Other components such as exercise guidance and nutritional advice were described as in the original studies to ensure completeness.

In this study, objective measurement refers to the use of wearable devices like accelerometers to directly record physical activity data, while subjective measurement refers to the assessment of physical activity levels through self-report questionnaires (e.g., PPAQ, IPAQ), which rely on participant recall. Based on this, among the 10 studies in the meta-analysis, assessment tools included subjective questionnaires in 6 studies and objective accelerometers in 4 studies. The Pregnancy Physical Activity Questionnaire (PPAQ) was the most commonly used subjective measure [30–32], followed by the International Physical Activity Questionnaire-Short Form (IPAQ-SF) [34, 37]. Objective measures included various brands of accelerometers such as Actigraph and activPAL3 [21, 33, 35, 36]. Regarding tool validation, two of the six studies using subjective questionnaires used the IPAQ-SF [34, 37] but did not mention specific validation in a pregnant population. For follow-up assessment, only one of the 16 studies included a post-intervention follow-up [33], which found no significant difference in physical activity levels between groups at 3 months postpartum. The other 15 studies did not report on the long-term effects after the intervention ended.

### Assessment of study quality and level of evidence

**Fig. 2:**
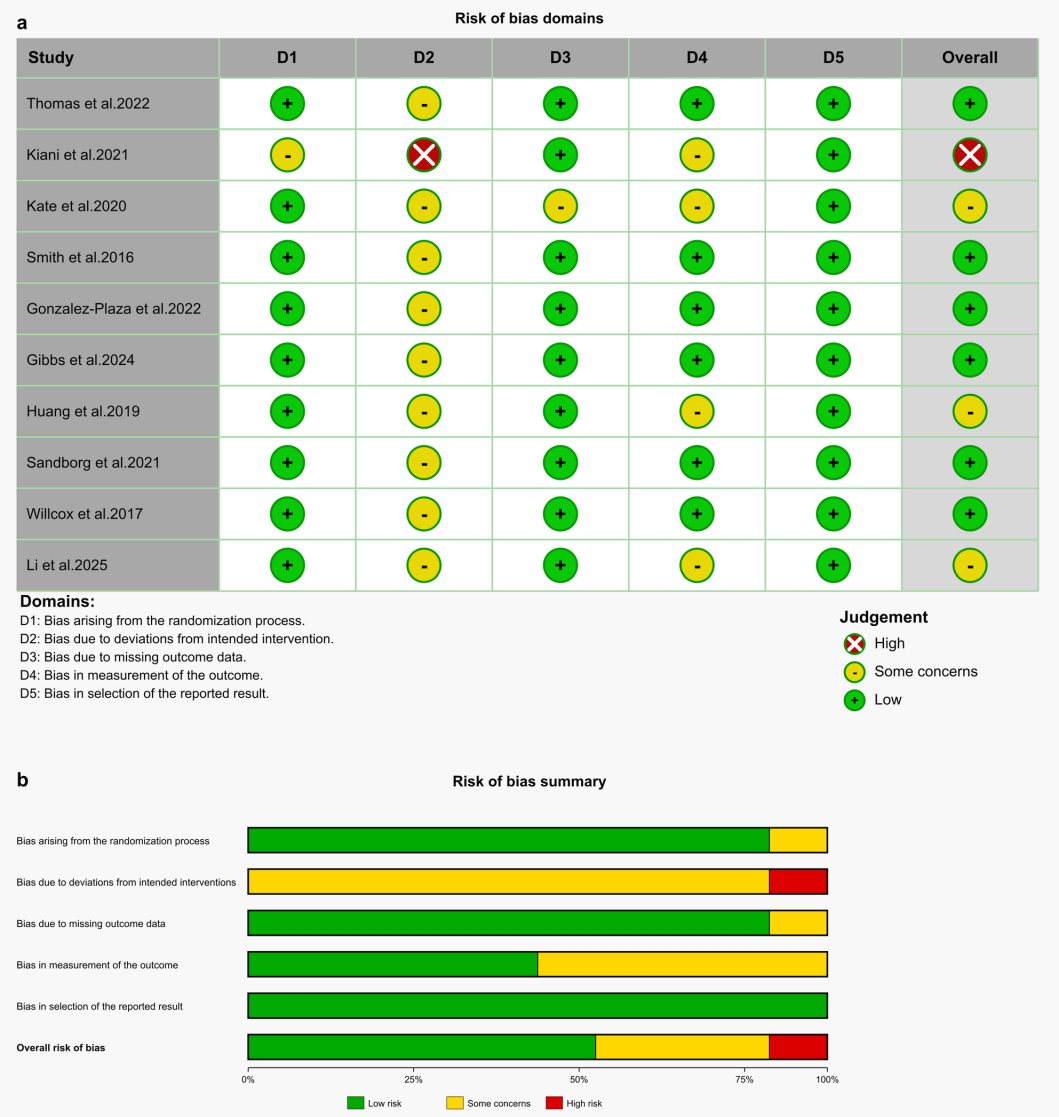
**a** Risk of bias summary: review of the authors judgments about each risk of bias item for each included study. **b** Risk of bias graph: review authors' judgments about each risk of bias item, presented as percentage of included studies

We first assessed the methodological quality of the 10 studies included in the meta-analysis using the Cochrane Risk of Bias tool 2.0 (RoB 2.0). The results indicated that most studies had a low risk of bias in the domains of the randomization process (D1), missing outcome data (D3), measurement of the outcome (D4), and selection of the reported result (D5). However, in the domain of deviations from intended interventions (D2), no study was rated as low risk, which was primarily attributed to the difficulty of blinding participants and personnel in behavioral intervention studies. Overall, approximately 60% of the studies were at low risk, 20% raised “some concerns,” and 10% were at high risk, suggesting that the overall methodological quality of the included studies was good (Fig. 2a and b).

Based on the quality assessment of individual studies, we further evaluated the overall quality of evidence for the main outcomes using the Grading of Recommendations Assessment, Development and Evaluation (GRADE) approach [44]. The GRADE evidence quality is classified into four levels: high, moderate, low, or very low. Two researchers independently completed the assessment, with the results detailed in Table 2.

**Table 2:** GRADE-Based evidence quality assessment for study conclusions

Study Outcome	Participants	GRADE Assessment Factors					Effect Size (95% CI)	Quality of Evidence
		Risk of Bias	Inconsistency	Indirectness	Imprecision	Other		
Moderate-to-Vigorous Physical Activity	859 (6RCT)	Serious	Not serious	Not serious	Not serious	Cannot assess	0.21 (0.07—0.34) I² = 36% *p* = 0.003	▢▢▢**○** Moderate
Total Physical Activit	974 (7RCT)	Serious	Serious	Not serious	Not serious	Cannot assess	0.44 (0.31—0.57) I² = 0% *p* < 0.0001	▢▢**○○**Low

A reliable assessment of publication bias could not be performed as the number of included studies was fewer than 10

### Meta-analysis results

#### Moderate-to-Vigorous physical activity (MVPA)

**Fig. 3:**
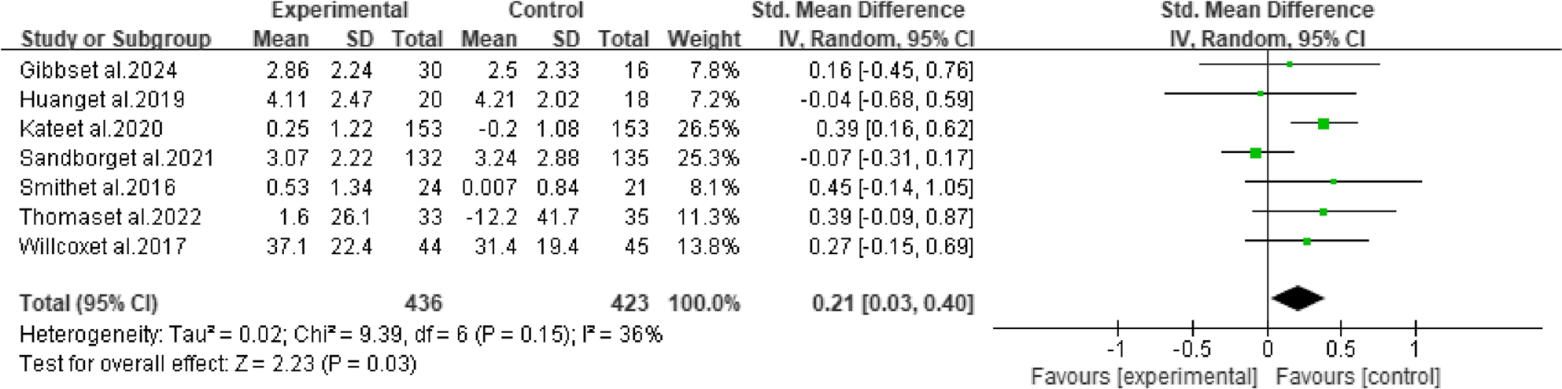
Meta-analysis results of moderate-to-vigorous physical activity

The meta-analysis of seven studies showed that mHealth interventions produced a small but statistically significant improvement in MVPA among pregnant women (SMD = 0.21, 95% CI: 0.03 to 0.40, *P* = 0.03), with moderate heterogeneity between studies (I² = 36%) (Fig. 3).

#### Total physical activity (TPA)

**Fig. 4:**
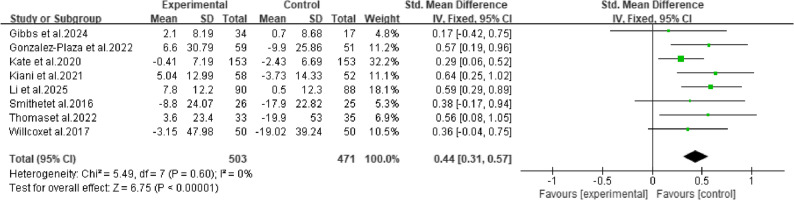
Meta-analysis results of total physical activity

The meta-analysis of eight studies showed that mHealth interventions produced a moderate and highly significant improvement in TPA among pregnant women (SMD = 0.44, 95% CI: 0.31 to 0.57, *P* < 0.00001), with very low heterogeneity (I² = 0%), indicating good consistency and robustness of the results (Fig. 4).

**Fig. 5:**
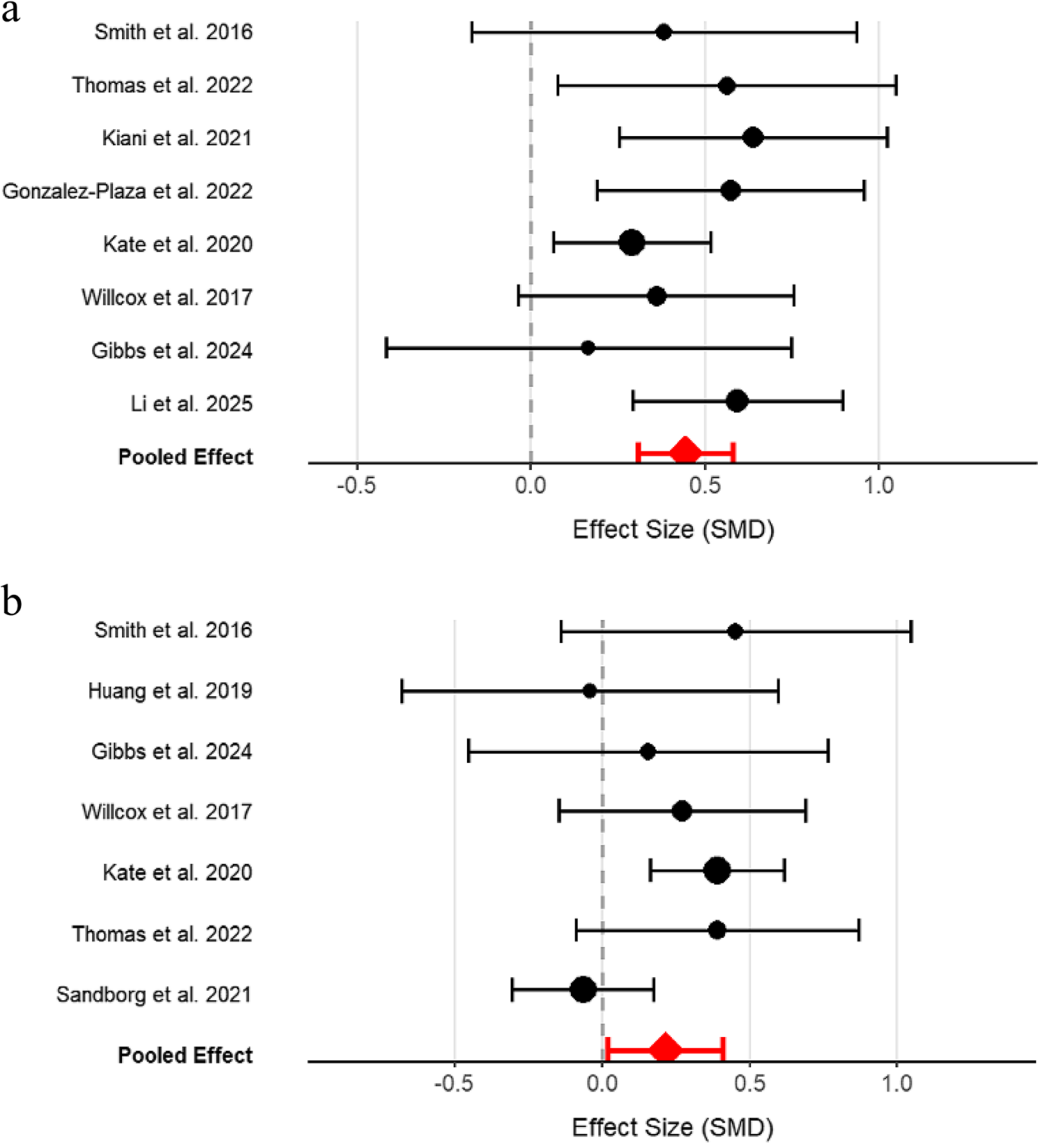
**a** Sensitivity analysis plot for MVPA (Moderate-to-Vigorous Physical Activity). **b** Sensitivity analysis plot for TPA (Total Physical Activity)

To assess the robustness of the meta-analysis results, we conducted a leave-one-out sensitivity analysis. The results showed that the findings for TPA were robust (Fig. 5a and b). In contrast, the sensitivity analysis for MVPA revealed greater variability. Removing the study by Sandborg et al. (2021) increased the effect size to 0.33 [0.17, 0.49] and reduced heterogeneity to 0%. Removing the study by Kate et al. (2020) reduced the effect size to 0.12 [−0.06, 0.30], losing statistical significance. The heterogeneity was primarily attributed to systematic differences in these two studies. The strong positive effect in Kate et al. (2020) was likely due to its large sample size, focus on a high-risk population (overweight/obese), and physical activity being the core intervention goal. Conversely, the negative effect in Sandborg et al. (2021) may reflect a dilution effect from including a large proportion of normal-weight women, the use of objective measures, and the study’s primary focus on gestational weight management rather than physical activity.

#### Exploratory meta-regression analysis

**Fig. 6:**
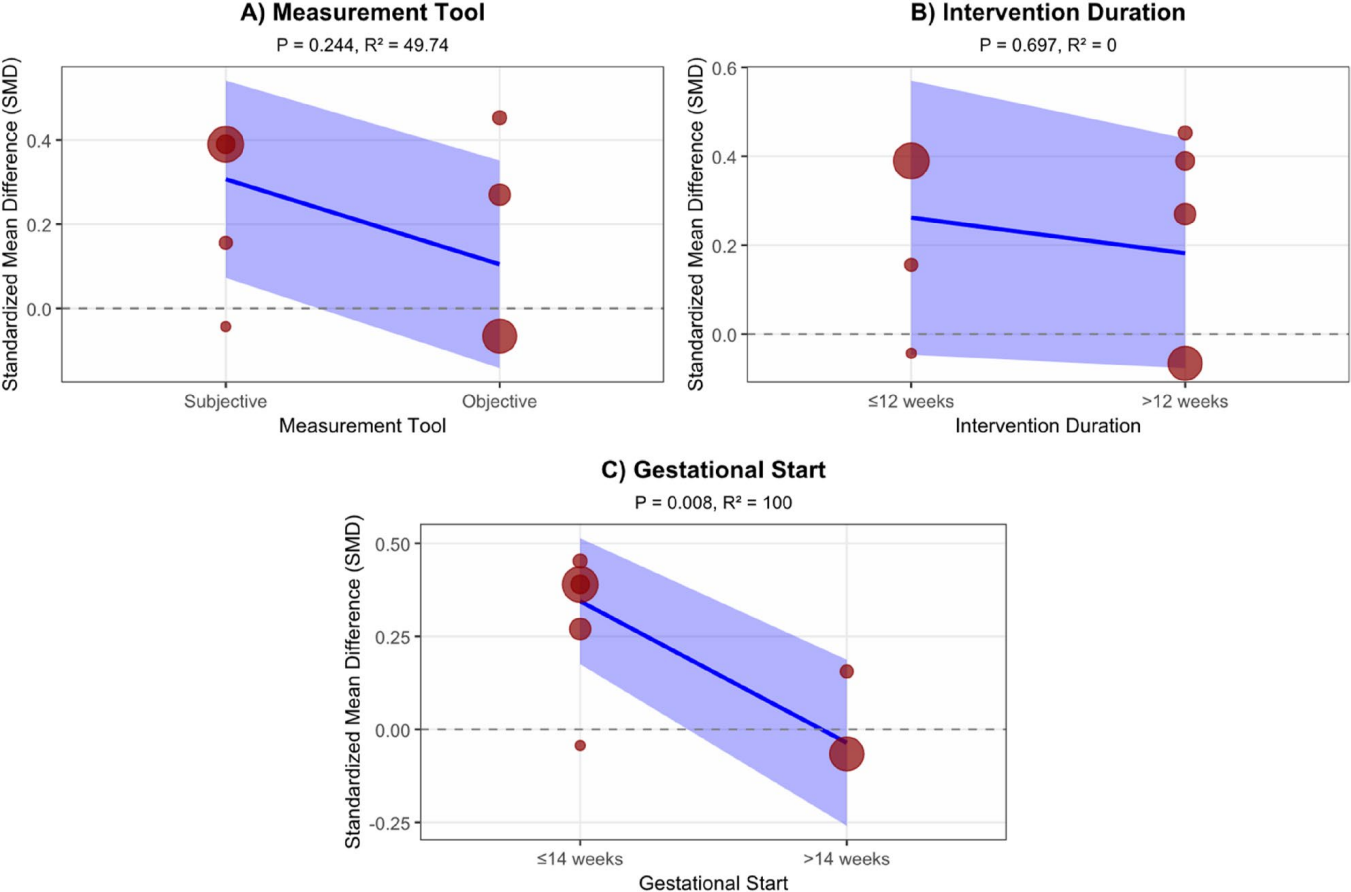
Exploratory meta-regression analysis results of moderate-to-vigorous physical activity. The three panels show the moderating effects of different variables on the intervention effect for MVPA (Moderate-to-Vigorous Physical Activity). Each scatter point represents an independent study, and the Y-axis represents the Standardized Mean Difference (SMD), reflecting the magnitude of the intervention effect. The blue solid line is the regression fit line, and the shaded purple area is the 95% confidence interval. **A** Moderating effect of measurement tool type (Subjective vs. Objective); **B** Moderating effect of intervention duration (≤ 12 weeks vs. >12 weeks); **C** Moderating effect of gestational start time (≤ 14 weeks vs. >14 weeks). The P-value indicates the statistical significance of the moderating effect, and R² represents the percentage of effect size variance explained by the moderator. The horizontal dashed line represents the no-effect line (SMD = 0)

Given the limited number of included studies, the following meta-regression analysis is exploratory. The analysis suggests that the gestational week at the start of the intervention may be an important factor influencing the effect on MVPA (*P* = 0.008, R² = 100%). Although the moderating effect of measurement tool type was not statistically significant (*P* = 0.244), its R² value of 49.74% suggests it may explain nearly half of the variance. Intervention duration did not show a moderating effect (*P* = 0.697, R² = 0%) (Fig. 6).

**Fig. 7:**
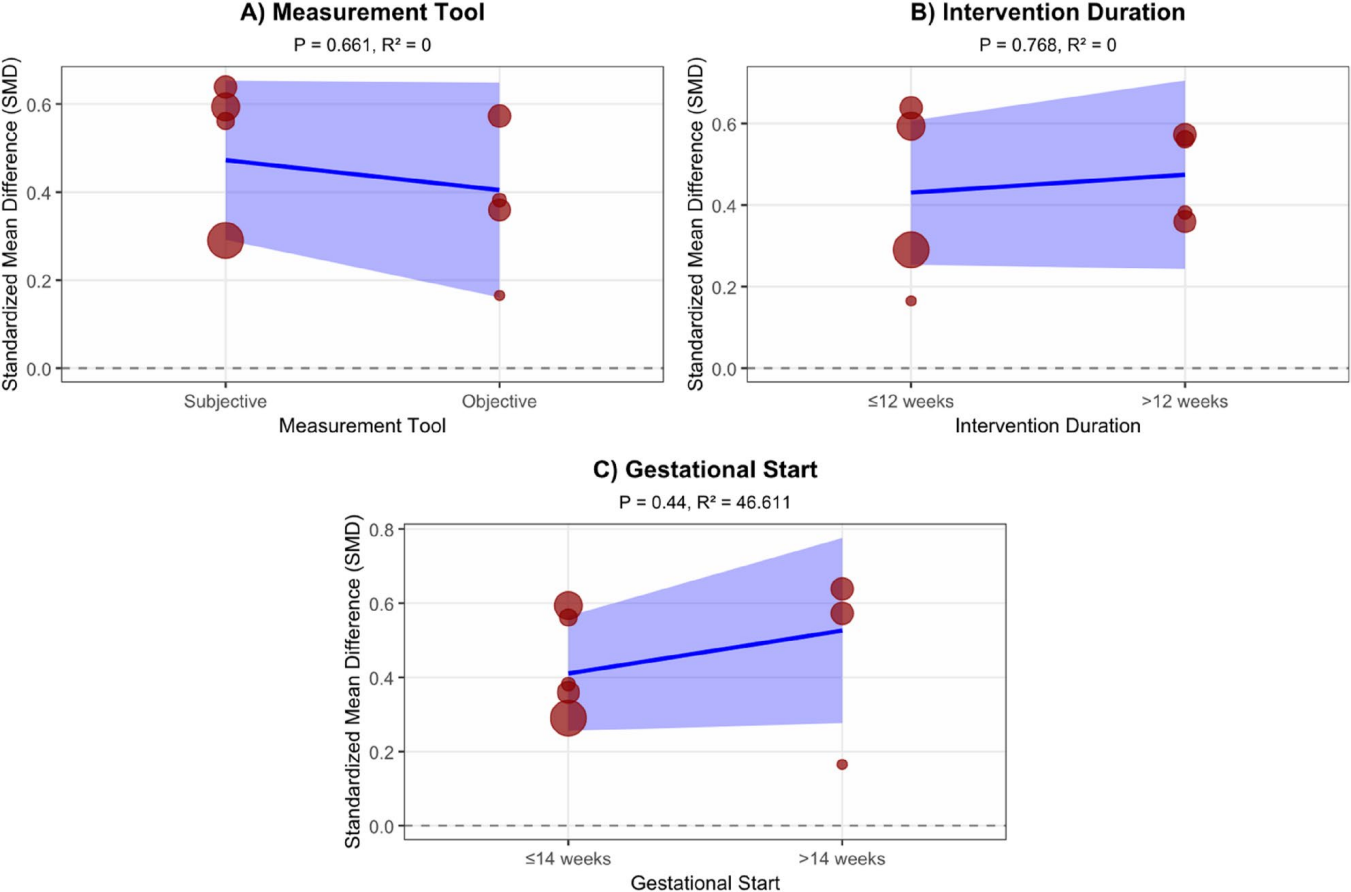
Exploratory meta-regression analysis results of total physical activity. The three panels show the moderating effects of different variables on the intervention effect for Total Physical Activity (TPA). Each scatter point represents an independent study, and the Y-axis represents the Standardized Mean Difference (SMD), reflecting the magnitude of the intervention effect. The blue solid line is the regression fit line, and the shaded purple area is the 95% confidence interval. **A** Moderating effect of measurement tool type (Subjective vs. Objective); **B** Moderating effect of intervention duration (≤ 12 weeks vs. >12 weeks); **C** Moderating effect of gestational start time (≤ 14 weeks vs. >14 weeks). The P-value indicates the statistical significance of the moderating effect, and R² represents the percentage of effect size variance explained by the moderator. The horizontal dashed line represents the no-effect line (SMD = 0)

The meta-regression analysis for Total Physical Activity (TPA) showed that none of the three moderators had a statistically significant effect on the intervention outcome. Although the moderating effect of gestational start time was not significant (*P* = 0.44), its R² value of 46.611% suggests that the difference in timing (≤ 14 weeks vs. >14 weeks) could still explain nearly half of the effect size variance. Neither measurement tool type (Subjective vs. Objective, *P* = 0.661, R² = 0%) nor intervention duration (≤ 12 weeks vs. >12 weeks, *P* = 0.768, R² = 0%) showed any moderating effect (Fig. 7).

#### Prespecified subgroup analysis

**Fig. 8:**
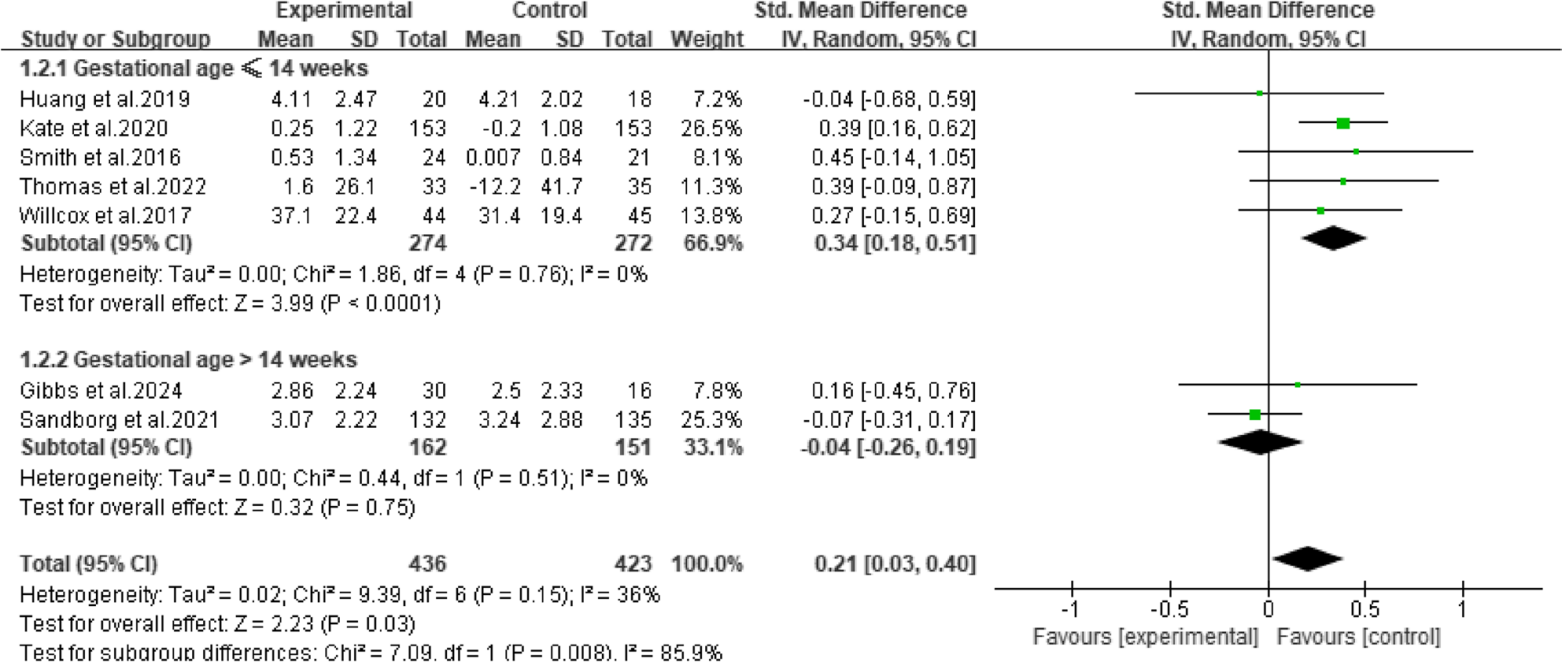
Prespecified subgroup analysis results of moderate-to-vigorous physical activity by gestational age at the initiation of intervention

Prespecified subgroup analysis showed that the gestational week at the start of the intervention was a significant factor for MVPA. The group starting at ≤ 14 weeks showed a significant positive effect (*P* < 0.0001) with no heterogeneity, while the group starting at > 14 weeks showed no significant effect (*P* = 0.75). The difference between subgroups was significant (*P* = 0.008) (Fig. 8). For other variables, although trends were observed (subjective measures showed larger effects than objective measures; shorter interventions showed larger effects than longer ones), these subgroup differences were not statistically significant. In contrast, the subgroup analyses for TPA showed robust effects across all subgroups, confirming that mHealth interventions are effective for increasing TPA regardless of these study characteristics.

### Publication bias

According to the Cochrane Handbook, when fewer than 10 studies are included, statistical methods such as funnel plots and Egger’s test have limited reliability. As the number of studies in our meta-analysis was limited (8 for TPA, 7 for MVPA), formal statistical tests for publication bias were not conducted. Although a comprehensive search strategy and sensitivity analysis were employed, the possibility of publication bias cannot be entirely excluded, particularly the potential non-publication of small studies or those with negative results. This is a potential limitation of the study and should be considered when interpreting the results.

## Discussion

### Summary of evidence

This study found that mHealth interventions have a positive but overall moderate effect on promoting physical activity in pregnant women. Specifically, the interventions produced a moderate and robust improvement in TPA (SMD = 0.44), while the effect on MVPA was relatively smaller (SMD = 0.21). It is noteworthy that the analysis of MVPA showed moderate heterogeneity (I² = 36%). Our sensitivity analysis revealed that this heterogeneity might primarily stem from clinical diversity among the included studies, particularly differences in study populations, the focus of the intervention, and outcome measurement tools. The prespecified subgroup analysis further indicated that MVPA and TPA might be regulated by different factors, reflecting the unique characteristics of different physical activity intensities.

### Comparison with previous studies and mechanistic analysis

The uniqueness of this study lies in its specific focus on the impact of mHealth interventions on physical activity during pregnancy, with a distinction between different activity intensities. Unlike previous reviews covering a broader range of health outcomes [45] or focusing on weight management [46], this study fills an evidence gap for this specific outcome. Our findings are highly consistent with a review by Singh et al. (2024) [47], which reported positive effects of digital health interventions on both TPA and MVPA, thus validating the reliability of our results. Notably, our findings differ from those of Sari et al. (2025) [23], which found no significant effect in a study exclusively on overweight/obese pregnant women. This discrepancy may reflect different responses to interventions across BMI populations and highlights the importance of intervention design and distinguishing between activity intensities.

We observed a potential moderating effect of measurement methods. The trend of subjective measures yielding larger effect sizes than objective measures has been reported in other populations [48] and is typically attributed to various biases in self-reporting [49] and the technical limitations of objective tools. From a mechanistic perspective, the success of mHealth interventions is primarily based on the integrated application of theories such as Social Cognitive Theory [50], the Transtheoretical Model [51], and Self-Determination Theory [52]. At the cognitive level, behavioral self-monitoring enhances users’ behavioral awareness and self-efficacy [53], enabling pregnant women to objectively understand their activity patterns and form accurate self-judgments. Behavioral feedback systems stimulate intrinsic motivation through immediate feedback [54], which aligns with the three basic psychological needs of competence, autonomy, and relatedness in Self-Determination Theory [55]. The goal-setting and progress-tracking functions enhance a sense of control by breaking down complex tasks through goal setting [56]. Additionally, social support networks are built through features like online communities, which are particularly important for pregnant women who require more understanding and encouragement from their social environment during this stage [57]. At the same time, the convenience of mHealth technology lowers participation barriers [58] and creates a more private intervention environment through technology mediation [59]. This study found that early-trimester interventions were more effective. The mechanism may be related to higher motivation for behavior change [60], lighter physical burden, and being in a critical period for habit formation during early pregnancy [61]. The observed advantage of short-term interventions (≤ 12 weeks) should be interpreted with caution. This phenomenon may not directly reflect that short-term interventions are inherently more effective but may instead reveal the inherent challenges faced by long-term interventions. As the intervention duration extends, the initial novelty and high motivation of participants may gradually wane [62], leading to decreased adherence and higher attrition rates, thereby diluting the overall effect of the long-term intervention [63]. Therefore, the significant effect of short-term interventions might simply capture data when participant adherence and motivation are at their peak. This possibility suggests that conclusions regarding intervention duration should be held with reservation in the absence of long-term follow-up data, and future research urgently needs to focus on how to maintain participant adherence in long-term interventions.

### Practical implications and evidence-based recommendations

This study provides important evidence for promoting physical activity during pregnancy. mHealth interventions yield statistically significant improvements, with a moderate effect on TPA (SMD = 0.44) and a small but consistent effect on MVPA (SMD = 0.21). It should be noted that a standard for the minimal clinically important difference (MCID) for physical activity improvement during pregnancy is currently lacking. Nevertheless, given that 70–80% of pregnant women do not meet WHO recommendations [64], these statistically significant improvements have potential practical value. Trends from our exploratory subgroup analyses suggest that early intervention (before 14 weeks) and short-term, intensive interventions (≤ 12 weeks) may be advantageous.

Based on the available evidence, we cautiously recommend considering mHealth interventions as a supplementary tool for promoting physical activity in antenatal care, especially for pregnant women with high technology acceptance. However, before clinical application, further research is needed to establish the MCID for physical activity improvement during pregnancy and to validate its association with maternal and infant health outcomes. To achieve this, the TIDIER checklist provides an important framework [65]. By using TIDIER to report intervention components in detail, future research can more clearly identify the crucial for achieving the MCID, thereby translating statistical significance into meaningful clinical improvements. Practitioners should remain prudent when applying these findings, recognizing the exploratory nature and limitations of the current evidence.

### Limitations and future directions

This study has several limitations. First, the limited number of studies included in the meta-analysis restricts the reliability of statistical analyses, including meta-regression and subgroup analyses. Second, the reliance on self-reported data in many studies introduces potential biases. Third, a general lack of long-term follow-up data limits our understanding of the sustainability of intervention effects. Fourth, some measurement tools used in the included studies have not been specifically validated for pregnant populations. Finally, the absence of an established MCID standard for physical activity in pregnancy makes the clinical significance of the observed effects difficult to ascertain.

To address these limitations, future research should focus on conducting large-scale, multicenter RCTs, developing and validating standardized physical activity assessment protocols for pregnant women, and designing studies with long-term follow-up. Additionally, research linking physical activity improvements to concrete maternal and infant health outcomes is needed to establish an MCID, and comprehensive analyses, including cost-effectiveness, should be conducted to provide robust evidence for policymakers.

## Conclusion

This systematic review evaluated the role of mHealth technology in improving physical activity during pregnancy. Specifically, the interventions produced a moderate and robust improvement in total physical activity (SMD = 0.44) and a small but significant positive impact on moderate-to-vigorous physical activity (SMD = 0.21). Subgroup analysis suggests that initiating the intervention in early pregnancy (≤ 14 weeks) may be more effective for promoting MVPA. These findings provide evidence-based support for using mHealth technology as an effective supplementary tool in routine antenatal care. However, given the limited number of included studies, these findings should be interpreted with caution, and future research in larger samples is needed for validation.

## Supplementary Information

Supplementary Material 1.

## Data Availability

The datasets supporting the conclusions of this meta-analysis are included in the article and its supplementary files. Search strategies and data extraction tables are available from the corresponding author upon reasonable request.
