# Supplementary material for: Effects of mobile health technology on physical activity in pregnant women: a systematic review and meta-analysis
Source: BMC Pregnancy Childbirth. 2025 Nov 21;25:1360. doi: 10.1186/s12884-025-08455-6 (PMC12751654; doi:10.1186/s12884-025-08455-6)
Supplement: Supplementary file 1 — Supplementary Material 1. [file 12884_2025_8455_MOESM1_ESM.docx]

Supplementary **Table 1** Basic Characteristics of Included Studies

| **Study Author/Publication Year** | **Subject Characteristics** | **Mean Age**  **(Experimental/Control Group)** | **Intervention Type**  **(Experimental/Control Group)** | **Intervention Components** | **Intervention Duration** | **Physical Activity Assessment Tool** | **Tool Assessment Content** |
| --- | --- | --- | --- | --- | --- | --- | --- |
| Thomas et al., 2022 | 68 (33/35) Mean BMI=28.9 kg/m² | 34.8±4.2 years / 33.2±3.7 years | Web-based mobile health intervention + telephone support / Routine prenatal care | Goal setting (BCT 1.1), self-monitoring (BCT 2.3), personalized feedback messages (BCT 2.2), wireless electronic scale, activity tracker, mobile health website, lifestyle coach telephone guidance | From 8-15 weeks gestation continued until before delivery, intervention lasted approximately 23 weeks, no follow-up | Pregnancy Physical Activity Questionnaire (PPAQ) | Uses Pregnancy Physical Activity Questionnaire (PPAQ) to assess 5 categories of activities over the past 2 months, light/moderate/vigorous intensity activity time, classified by METs |
| Kiani et al., 2021 | 93 (49/44) | 26.69 years / 25.15 years | Mobile application intervention / Routine prenatal care | Exercise benefits, exercise types, exercise plans, relaxation techniques, massage guidance, educational videos | From 16-20 weeks gestation continued until 28-32 weeks gestation, intervention lasted approximately 12 weeks, no follow-up | Pregnancy Physical Activity Questionnaire (PPAQ) | Persian version adapted from original PPAQ; measurement dimensions include total physical activity, MVPA, classified by METs |
| Kate et al., 2020 | 565 (278/287) Mean BMI=29.26±3.42 kg/m² | 32.84±4.6 years / 32.22±4.23 years | Mobile health lifestyle intervention / Routine prenatal care | Nutritional guidance, exercise prescription, mobile application, behavioral support | From 15-16 weeks gestation continued until 28 weeks gestation, intervention lasted approximately 12 weeks, no follow-up | Modified SLÁN 2002 Physical Activity Questionnaire | Adapted from Irish SLÁN 2002 national health survey questionnaire, categorized into light/moderate/vigorous intensity physical activity (frequency of 30-minute intervals per week), classified by METs |
| Smith et al., 2016 | 45 (24/21) Mean BMI=26.4±4.6 kg/m² | 29.7±4.1 years / 29.4±4.9 years | Web-based behavioral intervention / Routine prenatal care | Goal setting (BCT 1.1), self-monitoring (BCT 2.3), problem solving (BCT 1.2), interactive website based on social cognitive theory, community forum | From 10-14 weeks gestation continued until delivery, intervention period approximately 20-26 weeks, no follow-up | Accelerometer | Uses SenseWear Mini armband to record energy expenditure, physical activity intensity and duration, worn regularly during intervention period, with daily activity log |
| Gonzalez-Plaza et al., 2022 | 140 (78/72) BMI ≥30 kg/m² | 32.4±5.4 years / 33.4±4.7 years | Smart bracelet and midwifery counseling intervention / Routine prenatal care | Mi Band 2 smart bracelet, Mi Fit application, Hangouts application midwife health counseling | From 12-18 weeks gestation continued until 35-37 weeks gestation, intervention lasted approximately 21 weeks, no follow-up | International Physical Activity Questionnaire Short Form (IPAQ-SF) | Uses Spanish version of International Physical Activity Questionnaire Short Form, IPAQ-SF assesses walking, total physical activity, MVPA over past 7 days, classified by METs |
| Gibbs et al., 2024 | 51 (34/17) Mean BMI=28 kg/m² | 32.1±3.8 years | Multi-component sedentary behavior reduction integrated intervention / Routine prenatal care | Bi-weekly remote health coaching, wearable activity monitor, height-adjustable workstation, private Facebook group | From 14 weeks gestation continued until delivery or 38 weeks gestation, intervention lasted approximately 24 weeks, no follow-up | Accelerometer | Uses activPAL3 thigh accelerometer to measure standing time, walking time and daily steps, estimates moderate intensity activity. Worn for 8 days each in early/middle/late pregnancy, with diary to correct sleep periods, valid wear standard ≥5 days of valid data |
| Huang et al., 2019 | 57 (30/27) Mean BMI=25.7 kg/m² | 32.47±4.18 years / 34.15±4.02 years | Web-based mobile health intervention / Routine prenatal care | Goal setting (BCT 1.1), personalized feedback (BCT 2.2), dietary education, cognitive behavioral materials, one-on-one nutritionist consultation, weekly text contact, PLAN project website | From 8-11 weeks gestation continued until 18-23 weeks gestation, intervention lasted approximately 12 weeks. Follow-up period was 3 months postpartum, showing no significant difference in physical activity between groups | Accelerometer | Uses Actigraph wGT3X-BT three-axis accelerometer to record total PA and MVPA (classified by METs), worn for 7 days before and after intervention and at 36 weeks gestation, with wear diary to record non-wear time, valid wear standard ≥10 hours of waking wear per day |
| Sandborg et al., 2021 | 305 (152/153) 69.5% normal weight, 28.6% overweight/obese | 31.4±4.3 years / 31.3±3.8 years | Smartphone application intervention / Routine prenatal care | Goal setting (BCT 1.1), self-monitoring (BCT 2.3), personalized feedback (BCT 2.2), information education, push reminders, recipe guidance, exercise guidance | From 14 weeks gestation continued until 36 weeks gestation, intervention lasted approximately 23 weeks, no follow-up | Accelerometer | Uses Actigraph wGT3X-BT accelerometer to record moderate to vigorous intensity activity (classified by METs). Worn for 7 days each at baseline and 36 weeks gestation, sampling frequency 100Hz, analyzed using R software GGIR package, valid wear standard ≥10 hours of waking wear per day |
| Willcox et al., 2017 | 91 (45/46) Mean BMI=31.0±5.1 kg/m² | 33.0±3.4 years / 32.0±5.1 years | Multi-modal mobile health intervention / Routine prenatal care | Goal setting (BCT 1.1), self-monitoring (BCT 2.3), personalized text messages, mobile website, video messages, Facebook social platform, weight tracking, behavior change guidance | From 10-15 weeks gestation continued until 36 weeks gestation, intervention lasted approximately 23 weeks, no follow-up | Pregnancy Physical Activity Questionnaire (PPAQ) | Uses Pregnancy Physical Activity Questionnaire (PPAQ) to assess sedentary (<1.5 METs), light/moderate/vigorous intensity activity time over the past 2 months, classified by METs |
| Li et al., 2025 | 200 (100/100) Mean BMI ≥24 kg/m² | 30.49±3.99 years / 29.83±3.95 years | Mobile health technology lifestyle intervention / Routine prenatal care | Goal setting (BCT 1.1), self-monitoring (BCT 2.3), health education, physical activity intervention, WeChat public account | From 10-14 weeks gestation continued until 32-36 weeks gestation, intervention lasted approximately 12 weeks, no follow-up | International Physical Activity Questionnaire Short Form (IPAQ-SF) | Uses modified International Physical Activity Questionnaire Short Form (IPAQ-SF) referring to domestic modified validation |
| Knudsen et al., 2022 | 132 (87/45) Mean BMI=24.1 (IQR 21.8-28.7) kg/m² | 31.1±4.3 years / 32.0±4.6 years | Multi-modal mobile health intervention / Routine prenatal care | Goal setting (BCT 1.1), self-monitoring (BCT 2.3), personalized feedback messages (BCT 2.2), activity tracker | From ≤15 weeks gestation continued until delivery, intervention lasted approximately 26 weeks, no follow-up | Pregnancy Physical Activity Questionnaire (PPAQ) | Uses Danish version of Pregnancy Physical Activity Questionnaire (PPAQ-DK), participants digitally self-reported physical activity at 1st, 2nd, and 3rd visits, questionnaire assesses physical activity related to current pregnancy and daily activities |
| Chen et al., 2023 | 92 (46/46) 70.7% overweight, 29.3% obese | Experimental group ≤35 years: 67.4%; Control group ≤35 years: 76.1% | Multi-modal mobile health intervention / Routine prenatal care | Goal setting (BCT 1.1), self-monitoring (BCT 2.3), personalized feedback messages (BCT 2.2), activity tracker, mobile health website | From <17 weeks gestation continued until 34-36 weeks gestation, intervention lasted approximately 23 weeks, no follow-up | Pregnancy Physical Activity Questionnaire (PPAQ) | 24-hour recall questionnaire designed specifically for pregnant women, containing 32 activity items; covers total physical activity, moderate to vigorous physical activity (MVPA), classified by METs |
| Kathryn et al., 2014 | 33 (22/11) Mean BMI=28.5±4.5 kg/m² | 29±5 years / 32±2 years | Personalized text message intervention / Generic text message intervention | Goal setting (BCT 1.1), self-monitoring (BCT 2.3), personalized feedback (BCT 2.2), social support | From 12-21 weeks gestation continued until 32 weeks gestation, intervention lasted approximately 16 weeks, no follow-up | Pregnancy Physical Activity Questionnaire (PPAQ) | 24-hour recall questionnaire designed specifically for pregnant women, containing 32 activity items; covers total physical activity, moderate to vigorous physical activity (MVPA), classified by METs |
| Kozai et al., 2025 | 51 (34/17) Mean BMI=28.0±8.8 kg/m² | 31.7±4.7 years / 32.5±3.6 years | Physical activity increase intervention / Routine prenatal care | Bi-weekly individual behavioral coaching, social media groups, sit-stand workstation, wearable health tracker, reduced sitting time guidance, increased standing and walking guidance | From 10-12 weeks gestation continued until 32-34 weeks gestation, intervention lasted approximately 26 weeks, no follow-up | Accelerometer | Uses activPAL3 thigh accelerometer to measure standing time, walking time and daily steps, estimates moderate intensity activity. Worn for 7 days each in early/middle/late pregnancy, with diary to correct sleep periods |
| Choi et al., 2016 | 30 (15/15) Mean BMI=27.7±3.7 kg/m² | 32.9±2.5 years / 34.5±2.5 years | Mobile application + wearable device / Routine prenatal care | Goal setting (BCT 1.1), self-monitoring (BCT 2.3), automatic feedback (BCT 2.2), problem-solving skills training (BCT 1.2), daily message push, activity diary, face-to-face guidance | From 10-20 weeks gestation continued until 32 weeks gestation, intervention lasted approximately 12 weeks, no follow-up | Accelerometer | Wears Fitbit Ultra accelerometer to record daily activity types (such as brisk walking) and duration, distance, stairs climbed, estimated calorie consumption |
| Téoule et al., 2024 | 97 (49/48) 58% normal weight, 41% overweight/obese | 32±4 years | Comprehensive online health coaching intervention / Routine prenatal care | Professional pregnancy companion guidance, one-on-one video health coaching, application educational materials, fitness tracking device | From ≤20 weeks gestation continued until before delivery, intervention lasted approximately 24 weeks, no follow-up | Accelerometer | Wearable fitness tracking device with built-in motion sensor module, core function is recording daily activity steps, supports long-term wear and adapts to daily activity scenarios for pregnant women |


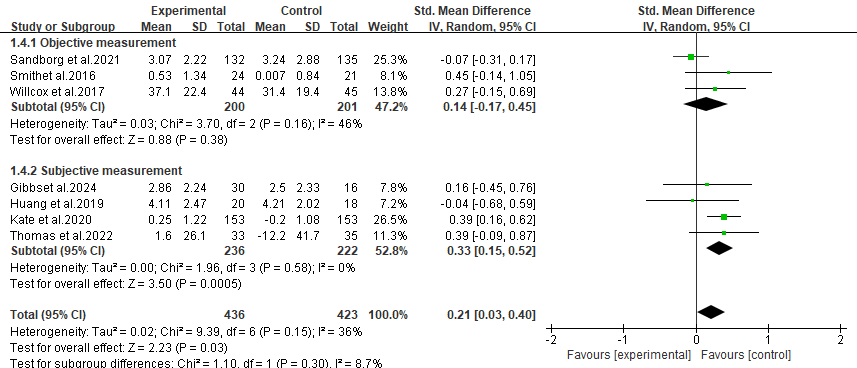


Supplementary Figure 1 Prespecified Subgroup Analysis Results of Moderate-to-Vigorous Physical Activity by Outcome Measurement Tool


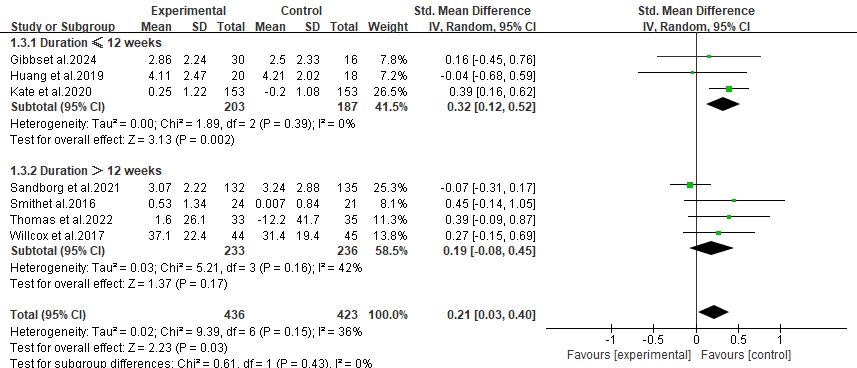


Supplementary Figure 2 Prespecified Subgroup Analysis Results of Moderate-to-Vigorous Physical Activity by Intervention Duration


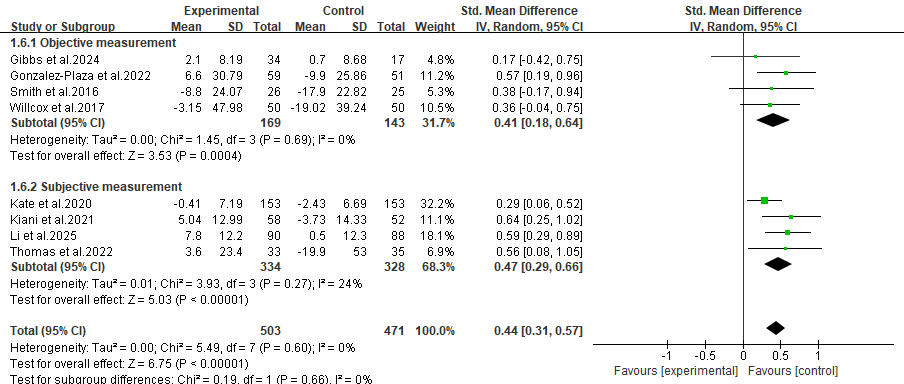


Supplementary Figure 3 Prespecified Subgroup Analysis Results of Total Physical Activity by Outcome Measurement Tool


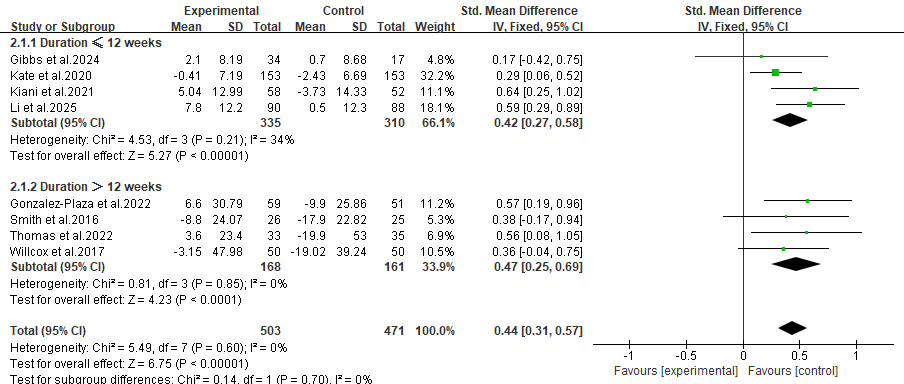


Supplementary Figure 4 Prespecified Subgroup Analysis Results of Total Physical Activity by Intervention Duration


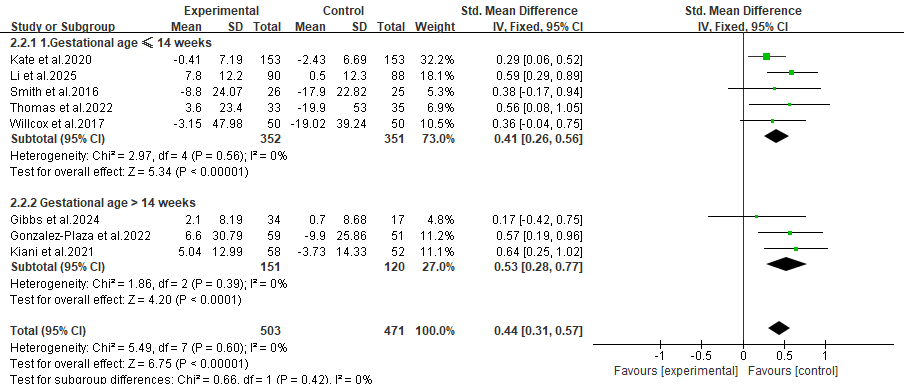


Supplementary Figure 5 Prespecified Subgroup Analysis Results of Total Physical Activity by Gestational Age at the Initiation of Intervention
